# Genetic and Functional Characterization of STAT4 in Rheumatoid Arthritis Patients with Distinct Disease Activity

**DOI:** 10.3390/ijms262010011

**Published:** 2025-10-15

**Authors:** Karla Mayela Bravo-Villagra, Rocio Guadalupe Hernández-Ruíz, Alejandra Landeros-Sáenz, Christian Johana Baños-Hernández, Sergio Cerpa-Cruz, Samuel García-Arellano, José Francisco Muñoz-Valle, Andres López-Quintero

**Affiliations:** 1 Instituto de Nutrigenética y Nutrigenómica Traslacional, Centro Universitario de Ciencias de la Salud (CUCS), Universidad de Guadalajara (UdeG), Guadalajara 44340, Mexico; karla.bravo2318@alumnos.udg.mx (K.M.B.-V.);; 2 Programa de Doctorado en Genética Humana, Centro Universitario de Ciencias de la Salud (CUCS), Universidad de Guadalajara (UdeG), Guadalajara 44340, Mexico; 3 Programa de Doctorado en Ciencias de la Nutrición Traslacional, Centro Universitario de Ciencias de la Salud (CUCS), Universidad de Guadalajara (UdeG), Guadalajara 44340, Mexico; 4 Antiguo Hospital Civil de Guadalajara “Fray Antonio Alcalde”, Guadalajara 44200, Mexico; 5 Instituto de Investigación en Ciencias Biomédicas, Centro Universitario de Ciencias de la Salud (CUCS), Universidad de Guadalajara (UdeG), Guadalajara 44340, Mexico

**Keywords:** *STAT4*, rheumatoid arthritis, rs7574865, rs11889341, pSTAT4, IL-12, IL-23, IFN-γ, mRNA

## Abstract

Rheumatoid arthritis (RA) is characterized by chronic inflammation mediated by the JAK/STAT pathway. Variants in *STAT4* have been associated with autoimmune susceptibility, but their functional role in RA remains unclear. The aim of this study was to genetically and functionally characterize *STAT4* in RA patients with varying disease activity by analyzing two variants, mRNA expression, phosphorylated STAT4 (pSTAT4), and inflammatory cytokines (IL-12, IL-23, and IFN-γ). Sixty-three Mexican patients with RA were stratified into remission/low and moderate/high activity groups. Genotyping, *STAT4* mRNA expression, pSTAT4 quantification, cytokine profiling, and treatment analyses were conducted. Patients receiving methotrexate, hydroxychloroquine, and sulfasalazine had higher IL-12 concentrations compared with those on other regimens. In remission/low activity patients, GC/GC carriers exhibited increased IL-12, PBMC levels, and anti-CCP antibodies, while GC/TT carriers in the moderate/high activity group showed distinct ESR values. Secondary analyses revealed that TT/TT carriers with *STAT4* overexpression exhibited higher IFN-γ and IL-23 levels. IL-12 differences persisted among GC/GC carriers regardless of *STAT4* expression status. In conclusion, these exploratory findings suggest potential interactions among *STAT4* haplotypes, expression status, and treatment regimens influencing cytokine and inflammatory profiles in RA. However, due to the small subgroup sizes, the observed associations should be interpreted with caution and considered hypothesis-generating until validated in larger cohorts.

## 1. Introduction

Rheumatoid arthritis (RA) is a chronic systemic inflammatory disease of autoimmune etiology that mainly affects the synovial joints, characterized by persistent inflammation of the synovial tissue, cartilage destruction, and bone erosion [1]. By 2020, it was estimated that 17.6 million people were living with RA, representing a 121% increase since 1990. In addition, the disease is more common in women than in men. In terms of geographical distribution, Latin America and the Caribbean have a prevalence rate of 269.7 [238.8 ± 306.6] per 100,000, ranking as the second region with the highest prevalence [2]. In Mexico, RA prevalence ranges between 1.6 and 2.8% of the general population, with higher frequency in women than in men [3,4]. Moreover, an increase in RA-related mortality, particularly among women, has been reported [5].

The pathogenesis of RA is heterogeneous and multifactorial, involving the complex interaction of genetic, environmental, and immunological factors [6]. Genome-wide association studies (*GWAS*) have identified more than 100 genetic susceptibility *loci* associated with RA, with major histocompatibility complex (*HLA*) genes exerting the greatest impact, particularly *HLA-DRB1* [7,8]. Nevertheless, non-*HLA* genes also contribute significantly to the risk of developing RA, including *PTPN22, TRAF1*, and notably *STAT4* [9,10]. The *STAT4* gene, located on chromosome 2q32.2-q32.3, encodes a 748-amino acid protein that plays a fundamental role in regulating both the innate and adaptive immune responses [11].

The STAT4 protein plays a crucial role in activating and regulating the adaptive immune response, mainly through the JAK/STAT (Janus kinase/signal transducer and activator of transcription) intracellular signaling pathway. This pathway is one of the most important routes for cytokine signal transduction, especially those involved in inflammatory and autoimmune processes [12,13,14]. STAT4 is primarily activated in the cytoplasm in response to cytokines such as interleukin-12 (IL-12), interleukin-23 (IL-23), and type I interferons, which bind to their specific receptors on the cell membrane and activate the associated tyrosine kinases JAK2 and TYK2. These kinases phosphorylate specific residues of STAT4, allowing its dimerization, translocation to the nucleus, and activation of target genes [10,15].

Within the nucleus, phosphorylated STAT4 induces the transcription of key genes such as interferon gamma (IFN-γ), an essential cytokine in the differentiation of T lymphocytes toward the Th1 phenotype, which plays a central role in the inflammatory response against intracellular pathogens [16,17,18,19]. STAT4 has also been implicated in the activation of Th17 cells, another subgroup of T lymphocytes that secrete IL-17, a cytokine associated with chronic inflammation and autoimmunity. Both Th1 and Th17 populations are widely recognized as key players in the pathophysiology of autoimmune diseases such as RA, where their dysregulation contributes to persistent joint damage [18,19].

The *STAT4* gene is highly conserved and generates multiple transcripts through alternative splicing, each performing different functions within the immune system. For example, STAT4α enhances IFN-γ secretion and antiviral responses, while STAT4β is more closely associated with cell proliferation and the perpetuation of chronic inflammation [20].

Several genetic studies have identified polymorphic variants of the *STAT4* gene, including rs7574865 and rs11889341, as susceptibility factors for RA and other autoimmune diseases such as systemic lupus erythematosus (SLE) [21,22,23,24]. These variants have been associated with increased gene expression or stronger activation of the STAT4 pathway, which could contribute to the chronic inflammatory state observed in these disorders. However, results in the literature have been inconsistent across populations, possibly due to genetic, environmental, or methodological differences.

The aim of this study was to genetically and functionally characterize *STAT4* in RA patients with different disease activity by analyzing two *STAT4* variants (rs7574865 and rs11889341) in relation to gene expression, phosphorylated protein levels, and inflammatory cytokines such as IL-12, IL-23, and IFN-γ. Evaluating these aspects in an integrated manner may provide a deeper understanding of the underlying immunological mechanisms in RA and contribute to the development of more specific therapeutic strategies.

## 2. Results

A total of 63 patients diagnosed with rheumatoid arthritis were included in the study and divided into two groups according to disease activity: group 1, remission-low (n = 31) and group 2, moderate-high (n = 32) (Table 1). No significant differences were observed between the groups regarding family history of autoimmune diseases (including RA, SLE, Crohn’s disease, or Sjögren’s syndrome), or lifestyle factors such as tobacco use (*p* > 0.05). Exposure to wood smoke was reported by 48.4% of patients in the remission-low group and 34.4% in the moderate-high group (*p* = 0.311). The mean age was similar between the groups (47.8 ± 11.75 vs. 50.25 ± 10.54 years, *p* = 0.389), as was the BMI (28.81 ± 6.55 vs. 27.40 ± 5.60 kg/m^2^, *p* = 0.364). The proportion of women was higher in both groups, without statistically significant differences (*p* = 0.104).

Differences were observed in erythrocyte count, hemoglobin, and hematocrit, which were lower in the moderate-high activity group (*p* < 0.01). No significant differences were found in leukocyte count, platelet count, or mean platelet volume between groups (see Supplementary Table S1).

Levels of C-reactive Protein (CRP), Rheumatoid Factor (RF), and anti-CCP antibodies were higher in the moderate-high group, although the differences were not statistically significant (*p* > 0.05). Erythrocyte Sedimentation Rate (ESR) was significantly higher in the moderate-high group (38.72 ± 8.29 mm/h vs. 23.29 ± 12.27 mm/h, *p* < 0.01).

Patients with moderate-high activity showed greater functional disability, as measured by the Health Assessment Questionnaire (HAQ) (1.14 ± 0.69 vs. 0.58 ± 0.64, *p* < 0.01), and had a higher number of tender and swollen joints (*p* < 0.01). Visual analog scale (VAS) scores for pain and the DAS28 disease activity score were also significantly higher in this group (*p* < 0.01). Limitation of movement was present in 53.1% of the moderate-high group compared to 12.9% of the remission-low group (*p* < 0.01).

Treatment regimens were generally similar across groups, with a statistically significant difference observed in hydroxychloroquine use: 35.5% in the remission-low group vs. 12.5% in the moderate-high group (*p* = 0.04).

Table 2 presents the comparison of inflammatory markers and *STAT4* gene expression, classifying patients according to disease activity (DAS28-ESR) into two groups: remission-low and moderate-high. No significant differences were observed for any of the variables (*p* > 0.05).

**Figure 1:**
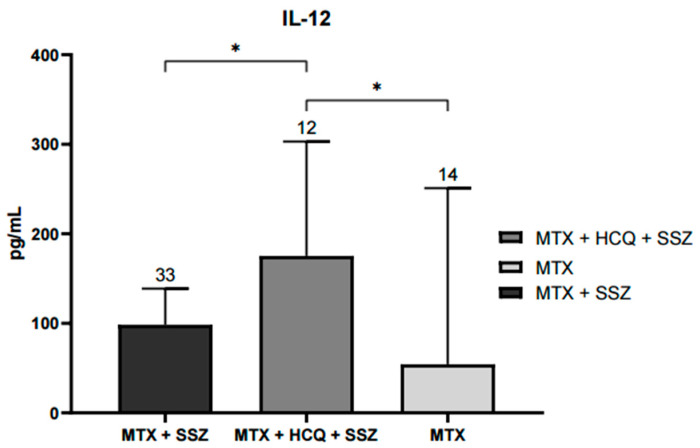
Serum IL-12 levels according to treatment schedule with disease-modifying antirheumatic drugs (DMARDs). Significant increases in IL-12 concentrations were observed in the group treated with MTX, HCQ, and SSZ compared with groups receiving MTX alone or MTX + HCQ (* *p* < 0.05). Data are expressed as median and interquartile range (pg/mL). Statistical differences were evaluated using the Kruskal–Wallis nonparametric test with Dunn’s post hoc comparison.

This study observed significant differences in serum IL-12 levels between treatment groups. Patients treated with the combination of methotrexate, hydroxychloroquine, and sulfasalazine (MTX + HCQ + SSZ) had significantly higher IL-12 concentrations compared with those receiving methotrexate alone or methotrexate plus hydroxychloroquine (*p* < 0.05). Four additional patients were not included in the graph because their treatment groups contained only a single individual each (one without treatment, one with sulfasalazine alone, and two with MTX + HCQ) and were therefore considered only descriptively (Figure 1).

The comparison of genotypes for the rs7574865 and rs11889341 variants of the *STAT4* gene between the remission-low and moderate-high activity groups (Table 3) showed no significant differences in genotype distribution. For the rs7574865 variant, genotypes GG, GT, and TT were observed in 36.7%, 48.4%, and 16.7% of the remission-low group, respectively, and in 28.1%, 50.0%, and 21.9% of the moderate-high group (OR for TT = 1.711, 95% CI = 0.403–7.271, *p* = 0.467). Similarly, the rs11889341 variant showed an identical genotype distribution (CC, CT, and TT) to rs7574865, without statistically significant differences (*p* > 0.05). Allele frequency analysis also revealed no significant associations, although both variants demonstrated a trend toward a higher T allele frequency in the moderate-high activity group.

**Figure 2:**
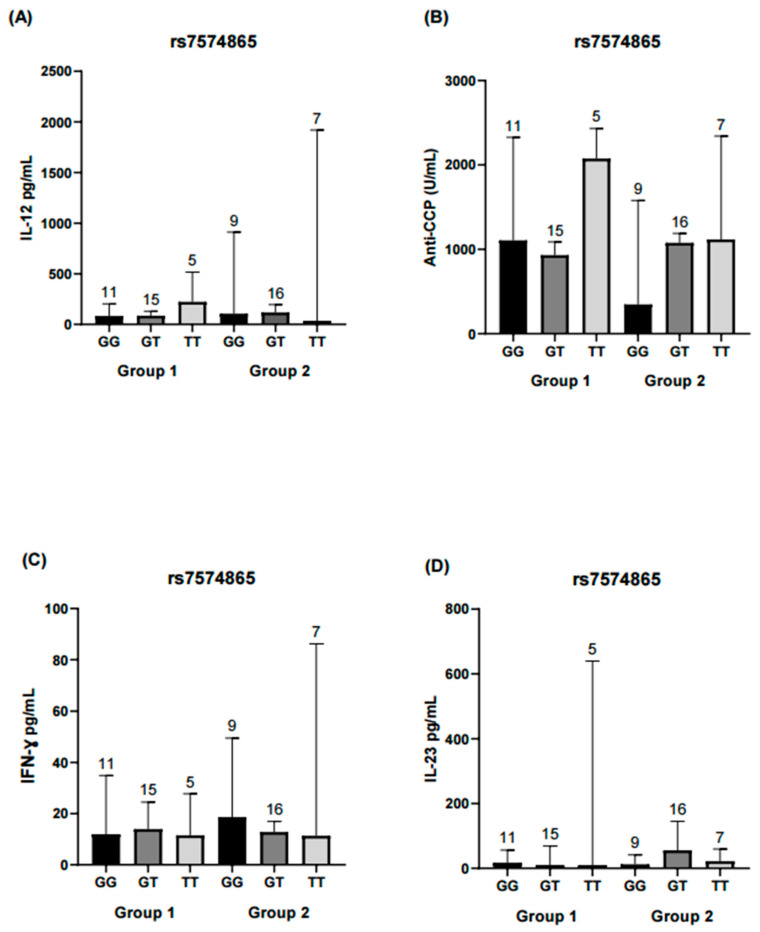
Comparison of IL-12, IL-23, IFN-γ, and anti-CPP levels across rs7574865 genotypes of the *STAT4* gene. Each bar represents a genotype (GG, GT, TT). Group 1 corresponds to RA patients in remission/low disease activity, and Group 2 corresponds to patients with moderate/high disease activity. (**A**) Serum IL-12 levels in both activity groups, showing no significant differences among genotypes. (**B**) Anti-CCP antibody concentrations in Group 2 were higher in TT and GT genotypes compared with GG, whereas in Group 1, higher values were observed in the TT genotype; however, differences were not statistically significant. (**C**) IFN-γ concentrations did not differ significantly between genotypes in either group. (**D**) IL-23 levels were similar across genotypes and did not reach statistical significance. Statistical comparisons were performed using the Kruskal–Wallis nonparametric test with Dunn’s post hoc comparison. Numbers above bars indicate the number of patients per genotype in each group.

Comparisons were made between patients grouped according to the genotypes of the rs7574865 variant and their level of disease activity to evaluate the distribution of various inflammatory markers. No significant differences were observed in IL-12, IL-23, or IFN-γ levels among the different genotypes analyzed. However, within the group with moderate-high disease activity (Group 2), carriers of the T allele showed higher concentrations of anti-CCP antibodies compared with non-carriers, although this difference did not reach statistical significance (Figure 2). This analysis was not repeated for the rs11889341 variant, as it exhibited the same genotypic distribution as rs7574865.

Haplotype analysis revealed that variants rs7574865 and rs11889341 were in strong linkage disequilibrium (D′ = 0.9997, r^2^ = 0.9997) (Table 4). No significant associations were identified between the TT or GC haplotypes and disease activity (*p* > 0.05). Subsequently, haplotype combinations were evaluated, forming the GC/GC, GC/TT, and TT/TT groups.

**Figure 3:**
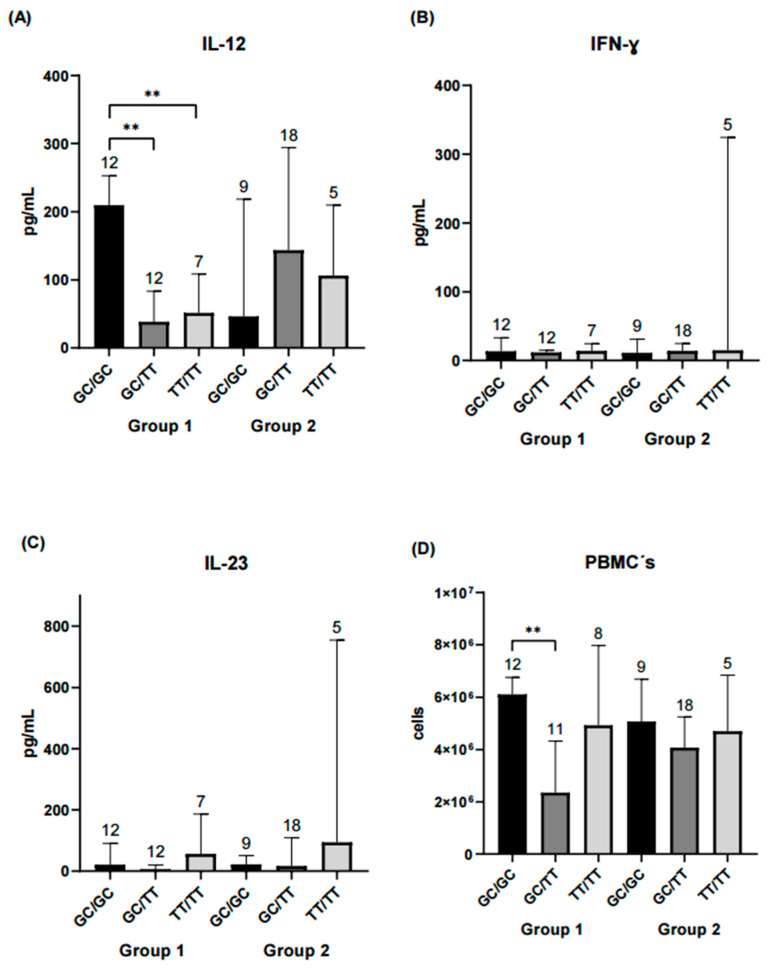
Comparison of IL-12, IFN-γ, IL-23, and PBMCs counts across *STAT4* haplotype combinations. Each bar represents a haplotype combination (GC/GC, GC/TT, TT/TT). Group 1 corresponds to RA patients in remission/low disease activity, and Group 2 corresponds to patients with moderate/high disease activity. (**A**) Serum IL-12 levels in both groups. Patients carrying the GC/GC haplotype combination in Group 1 exhibited significantly higher IL-12 concentrations compared with those with GC/TT or TT/TT (*p* < 0.01). No significant differences were observed among haplotype combinations in Group 2. (**B**) IFN-γ concentrations did not differ significantly between haplotype combinations in either group. (**C**) IL-23 levels were similar across haplotypes and did not reach statistical significance. (**D**) PBMC counts were significantly higher for the GC/GC haplotype compared with GC/TT in Group 1 (*p* < 0.01), whereas no differences were detected among haplotypes in Group 2. Statistical comparisons were performed using the Kruskal–Wallis nonparametric test with Dunn’s post hoc comparison. Numbers above bars indicate the number of patients per haplotype in each group. ** Significance value < 0.01.

**Figure 4:**
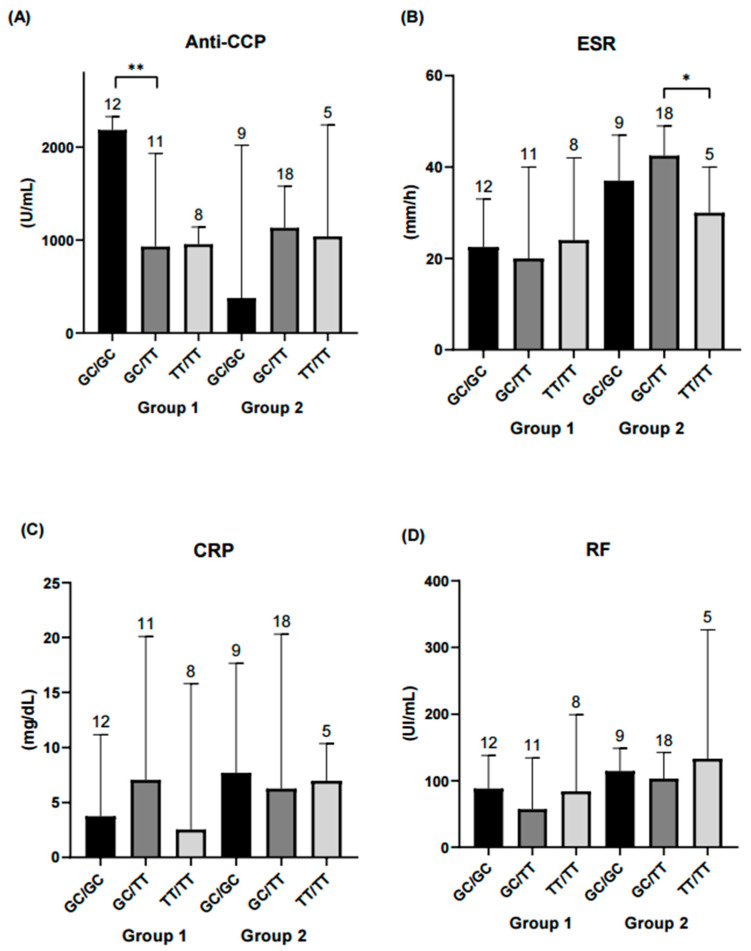
Comparison of diagnostic parameters across *STAT4* haplotype combinations. Each bar represents a haplotype combination (GC/GC, GC/TT, TT/TT). (**A**) Serum anti-CCP levels were significantly higher in the GC/GC haplotype combination within the remission-low activity group compared to GC/TT (*p* < 0.01). No significant differences were observed among haplotype combinations in the moderate-high activity group. (**B**) ESR was significantly higher in the GC/TT haplotype combination compared with TT/TT in the moderate-high activity group (*p* < 0.05). (**C**) CRP and (**D**) RF showed no significant differences among haplotype combinations. Statistical comparisons were performed using the Kruskal–Wallis nonparametric test with Dunn’s post hoc comparison. Numbers above bars indicate the number of patients per haplotype in each group. ** Significance value < 0.01; * Significance value < 0.05.

**Figure 5:**
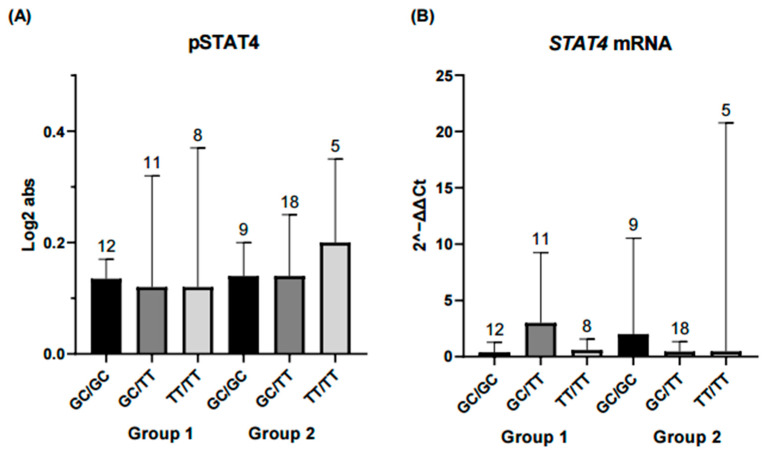
Comparison of STAT4 protein and gene expression across *STAT4* haplotype combinations. Each bar represents a haplotype combination (GC/GC, GC/TT, TT/TT). (**A**) Expression levels of phosphorylated STAT4 (pSTAT4) and (**B**) *STAT4* mRNA expression showed no significant differences among haplotype combinations in either the remission-low activity group or the moderate-high activity group. Basal pSTAT4 measurements were performed in non-stimulated PBMCs from patients receiving conventional DMARD therapy, which may influence basal phosphorylation and should be considered when interpreting these results. Statistical comparisons were performed using the Kruskal–Wallis nonparametric test with Dunn’s post hoc comparison. Numbers above bars indicate the number of patients per haplotype in each group.

The following analysis focused on evaluating haplotype combinations in relation to inflammatory and serological marker levels. In the remission-low activity group, higher concentrations of IL-12, peripheral blood mononuclear cells (PBMCs) and anti-CCP antibodies were observed in the GC/GC and GC/TT haplotype combinations. In contrast, in the moderate-high activity group, significant differences in ESR levels were observed between the GC/TT and TT/TT haplotype combinations (Figure 3, Figure 4 and Figure 5).

**Figure 6:**
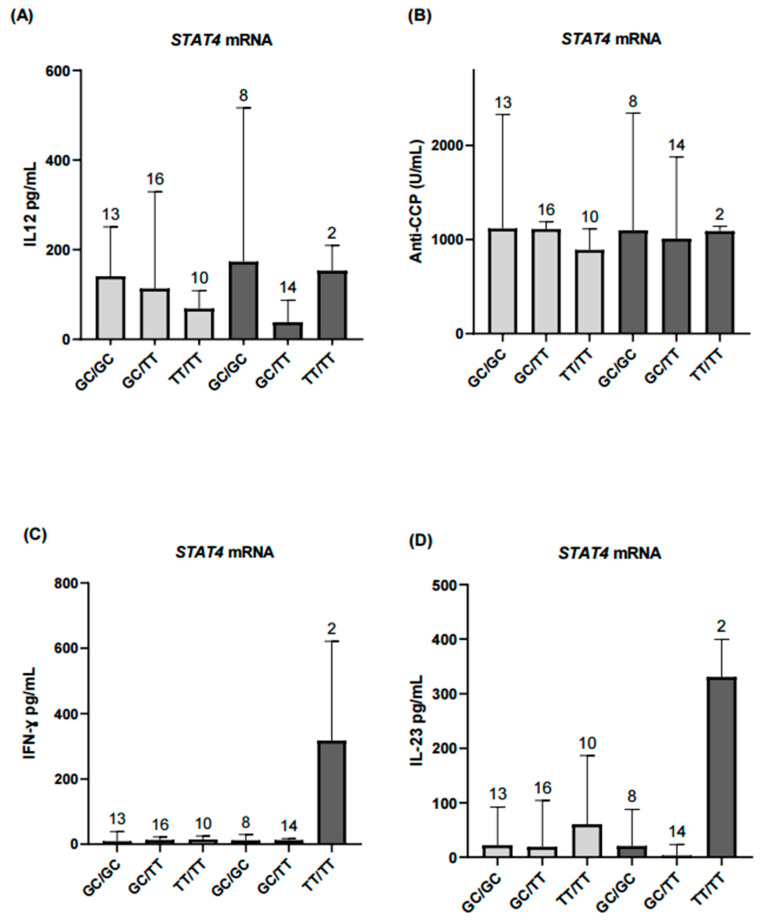
Comparison of inflammatory biomarkers according to *STAT4* mRNA expression levels. (**A**) Serum IL-12 levels showed no significant differences in the *STAT4* underexpression group (*p* > 0.05). (**B**,**C**) Anti-CCP antibody and IFN-γ levels were comparable in the underexpression group and did not reach statistical significance. (**D**) IL-23 levels showed no significant differences in the underexpression group. Light gray bars represent patients with *STAT4* underexpression, and dark gray bars represent patients with *STAT4* overexpression. Results for the overexpression group across all analyzed markers are presented descriptively and should be interpreted with caution due to the small sample size. Numbers above bars indicate the number of patients per haplotype in each group.

The main objective of the study was to compare levels of inflammatory and serological markers of disease activity in patients with RA according to genotype and haplotype. Secondary analyses considered the differential expression of *STAT4* mRNA, dividing the population according to expression status and comparing inflammatory and serological marker levels across haplotypes. TT/TT haplotype carriers exhibited higher IFN-γ and IL-23 levels in the overexpression group (Figure 6).

**Figure 7:**
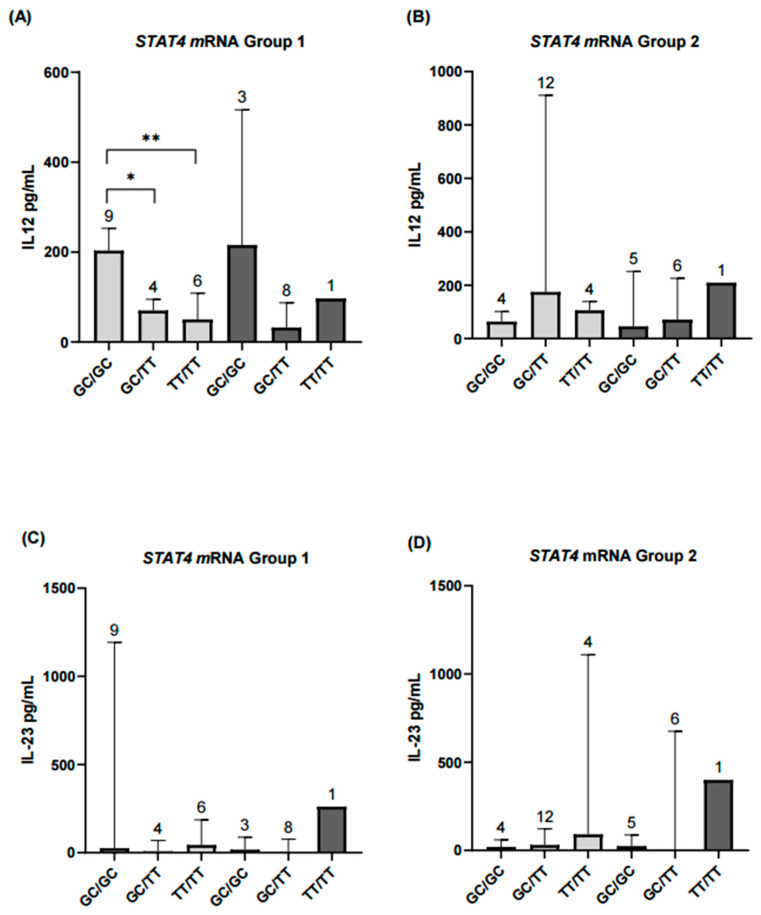
Comparison of serum IL-12 and IL-23 levels according to *STAT4* haplotypes and mRNA expression levels in rheumatoid arthritis patients with different disease activity. (**A**,**B**) In the remission–low activity group (Group 1), patients with *STAT4* underexpression and the GC/GC haplotype had significantly higher IL-12 levels compared with those carrying GC/TT (*p* < 0.05) or TT/TT (*p* < 0.01). In contrast, in the moderate–high activity group (Group 2), results for patients with *STAT4* overexpression are shown descriptively only and were not included in the statistical analyses. (**C**,**D**) For IL-23, patients with *STAT4* underexpression did not show significant differences between haplotypes (*p* = 0.07). Results from the *STAT4* overexpression subgroup in both activity groups are presented descriptively only. Numbers above bars indicate the number of patients per haplotype in each group. ** Significance value < 0.01; * Significance value < 0.05.

Continuing with the secondary exploratory analyses, patients were first grouped by disease activity and, within each group, further subdivided according to *STAT4* mRNA levels. IL-12 and IL-23 concentrations, considered biologically relevant in the context of autoimmune diseases, were then assessed. IL-12 levels differed significantly between patients in the remission-low activity group when comparing those with *STAT4* underexpression and overexpression. In contrast, no significant differences were observed in IL-23 levels between the groups defined by *STAT4* expression (Figure 7).

Spearman’s correlation analysis revealed significant associations among several variables. The DAS28 score showed a significant positive correlation with ESR (r = 0.70, *p* < 0.001), SSZ dose (r = 0.33, *p* < 0.05), and RF (r = 0.27, *p* < 0.05). MTX dose was positively correlated with SSZ dose (r = 0.44, *p* < 0.01). SSZ dose was also positively correlated with RF (r = 0.27, *p* < 0.05) and with IL-12 (r = 0.31, though this correlation did not reach statistical significance, *p* = 0.31). ESR showed a significant positive correlation with CRP (r = 0.30, *p* < 0.05). CRP was positively correlated with RF (r = 0.28, *p* < 0.05), while phosphorylated STAT4 protein (pSTAT4) was negatively correlated with RF (r = −0.27, *p* < 0.05).

**Figure 8:**
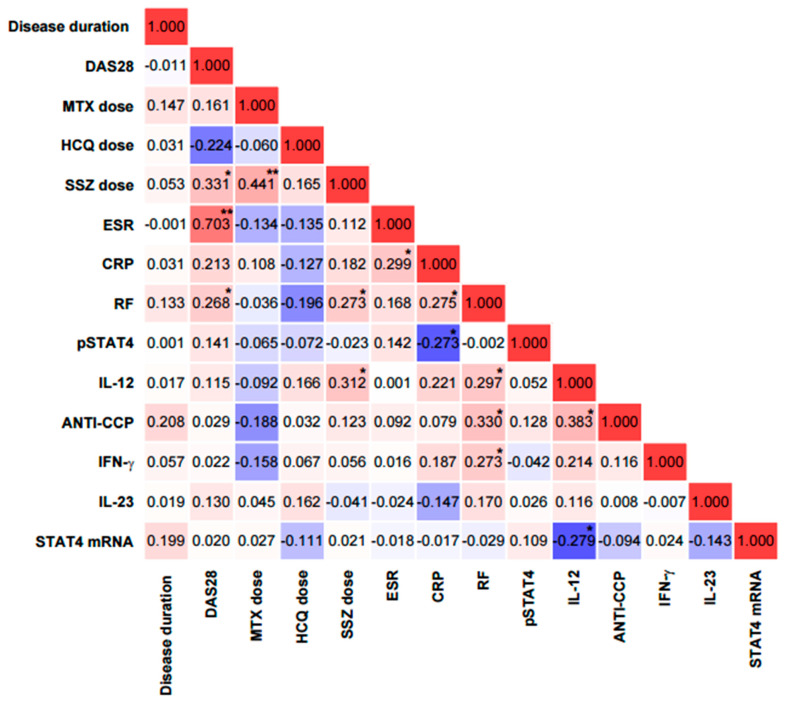
Correlation matrix between clinical, laboratory, and molecular variables in patients with rheumatoid arthritis. Correlation coefficients are color-coded: red indicates positive associations and blue indicates negative associations, with intensity reflecting the strength of the correlation. CRP = C-reactive protein; ANTI-CCP = anti-cyclic citrullinated peptide antibodies; RF = rheumatoid factor; ESR = erythrocyte sedimentation rate; DAS28 = disease activity score in 28 joints. ** Significance value < 0.01; * Significance value < 0.05.

In addition, RF was positively correlated with IL-12 (r = 0.30, *p* < 0.05), anti-CCP antibodies (r = 0.33, *p* < 0.05), and IFN-γ (r = 0.27, *p* < 0.05). IL-12 was also positively correlated with RF (r = 0.30, *p* < 0.05) and anti-CCP (r = 0.38, *p* < 0.05). Finally, IL-12 showed a negative correlation with *STAT4* mRNA expression (r = −0.28, *p* < 0.05) and a positive correlation with anti-CCP (r = 0.38, *p* < 0.05) (Figure 8).

## 3. Discussion

This study represents one of the first integrative molecular approaches to understanding the role of the JAK/STAT pathway, and specifically *STAT4* gene activity, in RA patients stratified according to disease activity. We evaluated the presence of two *STAT4* single nucleotide variants, gene expression (mRNA), phosphorylated STAT4 (pSTAT4) protein levels in PBMCs, and serum concentrations of IL-12, IL-23, and IFN-γ. This approach enabled us to assess how genetic and functional *STAT4* alterations may relate to immune activation and inflammatory status in RA [25].

The study population had a mean age of 48 years, with a higher proportion of women, consistent with the known epidemiology of RA [4]. The higher prevalence among women has been associated with hormonal changes, particularly reduced estrogen and progesterone levels during menopause or postpartum, which may influence disease risk and severity [26]. Biological sex substantially influences immune responses: females tend to mount stronger responses, which may increase their susceptibility to autoimmune diseases, whereas males are more vulnerable to acute viral infections and certain cancers [27,28]. In our study, however, only six patients were male, which precluded statistically meaningful sex-stratified analyses. As expected, clinical parameters used for disease classification differed significantly between groups, given that stratification was based on disease activity.

Despite these clinical differences, no significant differences were observed in MTX use between groups, aligning with the principle of treat-to-target (T2T) therapy, which is currently the standard approach for RA management. This model is based on the sustained reduction in inflammatory disease activity or, ideally, the achievement of clinical remission [29]. MTX is considered the benchmark drug among conventional synthetic DMARDs, due to its clinically proven efficacy and its recommendation as first-line treatment in international guidelines [30,31]. Interestingly, HCQ use was more frequent in the remission-low activity group. Although not recommended as monotherapy, HCQ has shown moderate benefit in early RA, particularly when administered in combination with other conventional synthetic DMARDs [32].

Triple therapy (MTX, HCQ, and SSZ) was the most common regimen, which is consistent with international recommendations, especially for patients with a suboptimal response to MTX alone. This combination has shown favorable pharmacodynamic synergy, associated with a significant improvement in disease control, particularly in cases of RA with less clinical aggressiveness [33]. Surprisingly, patients treated with triple therapy exhibited higher serum IL-12 concentrations, a finding that at first glance appears paradoxical. MTX has been shown to reduce IL12A expression in PBMCs in a dose-dependent manner [34]. However, IL-12 possesses a biphasic role (an early proinflammatory peak followed by a later anti-inflammatory phase), with proinflammatory and immunomodulatory functions depending on the context. In murine models, deletion of the IL-12 p35 subunit worsened inflammation, supporting this dual function [16]. This may explain our findings, suggesting that the IL-12/STAT4 axis could exert both pro- and anti-inflammatory effects depending on disease state and therapeutic context.

STAT4 is a key transcription factor in immune regulation, playing a central role in the JAK/STAT pathway. It is primarily activated by IL-12, and to a lesser extent, IL-23, promoting transcription of IFN-γ a type II interferon, along with other immune mediators [35,36]. Once phosphorylated, STAT4 translocates to the nucleus, driving Th1 and Th17 differentiation and amplifying inflammatory responses [37].

To date, several *STAT4* genetic variants have been identified, including rs7574865 and rs11889341, which are associated with an increased risk of developing rheumatoid arthritis [21,38,39,40]. In our cohort, individual analysis of these two *STAT4* SNVs did not reveal significant associations with inflammatory or immunological markers (anti-CCP, RF, CRP, PBMCs, IL-12, IL-23, IFN-γ), nor with disease activity. On the other hand, allele frequency distributions were consistent with previous reports in Mexican and Egyptian populations [21,22]. Notably, genetic associations with immune traits in RA and other autoimmune diseases have often produced inconsistent results across populations, likely due to varying environmental exposures, treatments, and genetic backgrounds [22,23,41].

In contrast, haplotype analysis revealed meaningful patterns. Although no differences in haplotype frequencies were observed between disease activity groups, specific combinations (particularly GC/GC) were associated with elevated IL-12 and PBMC counts among patients in remission or low disease activity. These findings reinforce the idea that combinations of variants may exert a more relevant biological effect than isolated variants, particularly in complex diseases with a multifactorial genetic basis such as RA, where gene–gene and gene–environment interactions are critical [10]. The lack of IL-12 differences in the moderate–high activity group may suggest that systemic inflammation and pharmacologic suppression mask genetic effects in more active disease states [41,42,43].

Interestingly, IL-23, and IFN-γ levels behaved similarly across different disease activity groups and were not associated with any haplotype combination. While these cytokines are recognized as central players in the pathogenesis of RA [17,19,36,41,42], their secretion is subject to highly dynamic and context-dependent regulation. Differences in cell subpopulations (e.g., CD8^+^ and CD4^+^ T cells), treatment effects, and immune-stromal interactions can modulate receptor expression, cytokine signaling, and downstream responses [41].

We also observed that the GC/GC haplotype was associated with higher PBMCs counts in patients with remission-low activity, suggesting a potential influence of the genetic profile on cellular balance in the immune response. In this regard, a previous study [44] linked changes in immune cell composition (such as increased CD4^+^ memory cells accompanied by reduced monocytes) with greater disease activity compared with healthy subjects, reinforcing the hypothesis that specific immune subpopulations may be directly related to the clinical progression of RA. On the other hand, anti-CCP levels were not significantly associated with the T allele of rs7574865, although a non-significant trend toward higher levels was observed in TT carriers, consistent with our previous findings [45]. When stratified by haplotype, anti-CCP concentrations were higher in the GC/GC carriers within the remission–low activity group, suggesting that antibody production may remain active despite clinically controlled inflammation. Taken together, these findings indicate that certain haplotype combinations could influence both disease activity and the patient’s immunological profile [10].

To further investigate *STAT4* function, we performed a secondary analysis based on *STAT4* mRNA expression status (under- vs. overexpression), independent of disease activity. Notably, patients with lower mRNA expression exhibited higher IL-12 levels, particularly in the GC/GC haplotype subgroup. This inverse relationship may reflect a compensatory regulatory mechanism in which IL-12 signaling is upregulated in response to decreased *STAT4* expression [46].

Earlier studies have reported an association between the T allele of the rs7574865 variant and increased *STAT4* expression (mRNA and total protein levels) [39], Similarly, Hagberg et al. demonstrated increased phosphorylated STAT4 and IFN-γ in remission SLE patients following in vitro stimulation of PBMCs with phytohemagglutinin (PHA)/IL-12 [41]. In contrast, we observed no significant differences in phosphorylated STAT4 levels between groups. This discrepancy likely reflects the different methodological approaches: while stimulation assays evaluate the transient activation capacity of the JAK/STAT pathway [47], our design aimed to characterize basal (non-stimulated) STAT4 phosphorylation in RA patients under different DMARD therapies. Both strategies provide complementary insights, stimulation reveals the dynamic signaling potential, whereas basal measurements capture the immune status under real therapeutic conditions. The basal characterization of PBMCs may reveal biological and treatment-related differences. Therefore, these findings underscore the importance of interpreting null results in pSTAT4 phosphorylation with caution, rather than indicating a true absence of biological effect, they likely reflect the experimental design and the pharmacological modulation of the pathway.

This study provides insights into *STAT4* heterogeneity by comparing RA patients with different disease activity. However, progressive stratification into small subgroups may have limited statistical power, and pSTAT4 measurements under basal conditions in patients receiving DMARD therapy may not fully capture the transient dynamics of the JAK/STAT pathway. While a healthy control group was not included, its incorporation in future studies could help assess subtle differences and provide broader context. Overall, these results should be considered exploratory and require validation in studies with larger sample sizes.

Nevertheless, an important strength of this work is the integration of genetic, transcriptional, and protein data under baseline (non-stimulated) conditions in clinically well-characterized RA patients, providing valuable insights into immune regulation.

## 4. Materials and Methods

### 4.1. Study Population

This study included 63 Mexican patients diagnosed with RA, originating from western Mexico and recruited from the outpatient Rheumatology Department of Hospital Civil “Fray Antonio Alcalde” in Guadalajara, Jalisco. Participants were classified into two groups based on disease activity: the first group included patients in remission or with low activity, and the second group included those with moderate or high disease activity, according to the Disease Activity Score 28 (DAS28). Importantly, PBMCs from all patients were analyzed under basal (non-stimulated) conditions for the measurements performed in this study.

Clinical and demographic variables were recorded, including disease duration, swollen and tender joint counts, VAS for pain, HAQ, and pharmacological treatment. The diagnosis of RA was established according to the 2010 ACR/EULAR classification criteria. Relevant serological biomarkers were also assessed, including RF, anti-CCP, CRP, and ESR.

All participants provided written informed consent prior to inclusion in the study. Patients with overlapping rheumatic diseases such as SLE, Sjögren’s syndrome, systemic sclerosis, ankylosing spondylitis, psoriatic arthritis, fibromyalgia, Behçet’s syndrome, or gout, as well as those under biologic treatment, were excluded. The study protocol was approved by the Bioethics Committee of the Centro Universitario de Ciencias de la Salud (CUCS), Universidad de Guadalajara (CI-03723), and by the Ethics Committee of Hospital Civil “Fray Antonio Alcalde” (CEI 251/23).

### 4.2. Serological and Immunological Parameters

Serum levels of anti-CCP antibodies were measured using an ELISA assay (REF EA1505–9601G; EUROINMUN, San Sebastián de los Reyes, Madrid, Spain), with a positivity cutoff of >5 U/mL. RF (IU/mL) and CRP (mg/L) were quantified by turbidimetric methods (codes 31922 and 31921, respectively; BioSystems, Barcelona, Spain). ESR (mm/h) was determined using the classical Wintrobe method. All serological and biochemical analyses were conducted at the Institute for Biomedical Research (IICB) and the Translational Institute of Nutrigenetics and Nutrigenomics (INNUGET) of the Universidad de Guadalajara.

### 4.3. Proinflammatory Cytokines

Serum concentrations of IL-12 (pg/mL) were determined using the ProQuantum™ Human IL-12p70 Immunoassay Kit (Thermo Fisher Scientific, Waltham, MA, USA), an ultrasensitive assay based on qPCR amplification. IL-23 and IFN-γ concentrations (pg/mL) were measured using sandwich-type ELISA kits (Thermo Fisher Scientific, Waltham, MA, USA). All assays were performed according to the manufacturer’s instructions, using serum samples previously stored at −80 °C.

### 4.4. Genotyping of STAT4 Variants

Genomic DNA from 63 patients was genotyped by allelic discrimination assays using predesigned TaqMan^®^ probes for the rs7574865 (ID: C_29882391_10) and rs11889341 (ID: C_26419582_10) variants (Applied Biosystems, Foster City, CA, USA). Genotyping was performed by allele-specific fluorescence detection using real-time PCR on the LightCycler^®^ system (Roche, Barcelona, Spain).

### 4.5. STAT4 mRNA Expression

*STAT4* mRNA expression was evaluated in PBMCs from all 63 patients. Total RNA was extracted using TRIzol^®^ Reagent (Invitrogen, Carlsbad, CA, USA) and quantified with a Multiskan™ spectrophotometer controlled by SkanIt 7.0 software (Thermo Fisher Scientific). Only samples with an A260/280 ratio between 1.8 and 2.1 were included. Complementary DNA (cDNA) was synthesized from 1 µg of total RNA using M-MLV Reverse Transcriptase (Promega, Madison, WI, USA).

Gene expression was quantified by real-time PCR on a LightCycler^®^ system (Roche, Barcelona, Spain) using TaqMan^®^ probes specific for *STAT4* and reference genes *GAPDH* (Hs02758991_g1) and *ACTB* (Hs01060665_g1). Each reaction was conducted in a final volume of 5 µL under the following conditions: initial denaturation at 95 °C for 10 min, followed by 40 cycles at 95 °C for 15 s and 60 °C for 1 min. All samples were analyzed in duplicate. Relative *STAT4* expression was calculated using the 2^–ΔΔCt^ method, with the control group mean as calibrator.

### 4.6. Semiquantitative Detection of pSTAT4

Protein was extracted from PBMCs isolated by density gradient centrifugation using Lymphoprep™ (Stemcell Technologies, Vancouver, BC, Canada). Cells were lysed on ice with RIPA buffer (Thermo Fisher Scientific, Cat. No. 89900), supplemented with a protease and phosphatase inhibitor cocktail (Sigma-Aldrich, St. Louis, MO, USA) to preserve phosphorylation. After 30 min of gentle agitation, lysates were centrifuged at 13,000 rpm for 15 min at 4 °C, and the supernatant was collected.

Phosphorylated STAT4 (pSTAT4) was detected using the Human Phospho-STAT4 (Tyr693) ELISA Kit (ab246529; Abcam, Cambridge, UK). Absorbance was measured at 450 nm with a Multiskan™ microplate reader (Thermo Fisher Scientific), operated with SkanIt 7.0 software. All samples were analyzed in duplicate.

### 4.7. Statistical Analysis

Genotype and allele frequencies of rs7574865 and rs11889341 were obtained by direct counting and compared using the chi-square test. Linkage disequilibrium analysis was performed with HaploView, and haplotype combinations were generated in R (RStudio) (version 2024.12.1+563).

Normality of continuous variables was assessed using the Shapiro–Wilk test. Student’s *t*-test was applied to compare means between two groups, and the Mann–Whitney U test was used for medians. For comparisons among more than two groups of nonparametric data, the Kruskal–Wallis test was applied, followed by Dunn’s multiple comparison test with Bonferroni adjustment, as implemented in GraphPad Prism 8. All reported *p*-values correspond to multiplicity-adjusted results.

Differences between genotypes, allele frequencies, haplotypes, clinical scores (HAQ, VAS), lifestyle variables, family history, mRNA expression levels, and pSTAT4 concentrations were analyzed using the chi-square test in R (RStudio) and GraphPad Prism 8. In addition, binary logistic regression was applied to assess associations between genetic variants and clinical disease activity. Results were expressed as odds ratios (OR) with 95% confidence intervals (95% CI). A *p*-value < 0.05 was considered statistically significant.

## 5. Conclusions

Our findings indicate that treatment influences cytokine profiles, as patients receiving methotrexate in combination with hydroxychloroquine and sulfasalazine exhibited elevated IL-12 levels compared with other regimens. The GC/GC haplotype was associated with higher IL-12 levels, PBMC counts, and anti-CCP antibodies in patients with remission or low disease activity, whereas the GC/TT haplotype was linked to differences in ESR among those with moderate to high activity.

Exploratory analyses incorporating *STAT4* mRNA expression further revealed that TT/TT haplotype carriers with overexpression displayed increased IFN-γ and IL-23, while IL-12 differences among GC/GC carriers persisted regardless of expression status. Taken together, these results suggest potential interactions between *STAT4* haplotypes, gene expression, and therapeutic context in RA. However, given the reduced sample sizes of certain subgroups, these associations should be interpreted with caution and regarded as hypothesis-generating until validated in larger, independent cohorts.

## Figures and Tables

**Table 1:** Clinical, anthropometric, and treatment characteristics by disease activity in patients with rheumatoid arthritis.

Variable	Remission-Low Group (n = 31)	Moderate-High Group (n = 32)	*p*-Value
Family history and lifestyle factors			
Rheumatoid arthritis +	17 (54.8)	14 (43.8)	0.454 ^c^
Type 1 Diabetes +	11 (35.5)	9 (28.1)	0.595 ^c^
Crohn’s disease	2 (6.5)	1 (3.1)	0.613 ^c^
Systemic lupus erythematosus +	2 (6.5)	2 (6.3)	1.00 ^c^
Multiple sclerosis	0 (0)	2 (6.3)	0.492 ^c^
Sjögren’s syndrome +	2 (6.5)	0 (0)	0.238 ^c^
Tobacco use	5 (16.1)	10 (31.3)	0.237 ^c^
Years smoking	0 (0–30)	0 (0–62)	0.224 ^b^
Cigarettes per day	0 (0–7)	0 (0–20)	0.111 ^b^
Wood smoke exposure +	15 (48.4)	11 (34.4)	0.311 ^c^
Years exposed	0 (0–60)	0 (0–42)	0.183 ^b^
Anthropometric data			
Age	47.80 ± 11.75	50.25 ± 10.54	0.389 ^a^
Female	26 (83.9)	31 (96.9)	0.104 ^c^
Male	5 (16.1)	1 (3.1)	
BMI (kg/m^2^)	28.81 ± 6.55	27.40 ± 5.60	0.364 ^b^
Clinical evaluation			
Disease duration (years)	5.0 (0.08–29)	3 (1–16)	0.566 ^b^
HAQ	0.58 ± 0.64	1.14 ± 0.69	<0.01 ** **^a^**
Tender joints	0 (0–3)	2 (0–16)	<0.01 ** **^b^**
Swollen joints	0 (0–4)	1 (0–7)	<0.01 ** **^b^**
ESR (mm/h)	23.29 ± 12.27	38.72 ± 8.29	<0.01 ** ^a^
VAS (Patient)	27.1 ± 19.87	61.25 ± 18.09	<0.01 ** **^a^**
DAS28	2.58 (1.64–3.2)	4.29 (3.22–6.65)	<0.01 ** **^b^**
Morning stiffness +	15 (48.4)	21 (65.6)	0.207 **^c^**
Duration of stiffness (min)	0 (0–180)	7.50 (0–120)	0.206 ^b^
Movement limitation +	4 (12.9)	17 (53.1)	<0.01 ** ^c^
PBMCs	4.43 × 10^6^ ± 2.16 × 10^6^	4.15 × 10^6^ ± 1.79 × 10^6^	0.580 ^a^
CRP (mg/dL)	3.78 (0.65–24.32)	7.38 (1.77–75.85)	0.102 ^b^
RF (IU/mL)	78.1 (0.0–287.60)	113.63 (2.66–490.30)	0.105 ^b^
Anti-CCP (U/mL)	1076 (0.0–2445)	1078 (0.0–2343)	0.778 ^b^
Treatment type			
Celecoxib +	22 (71)	24 (75)	0.782 ^c^
Celecoxib dose (mg)	200 (0–400)	200 (0–400)	0.781 ^b^
Alendronate +	5 (16.1)	4 (12.5)	0.732 ^c^
Alendronate dose (mg)	0 (0–10)	0 (0–10)	0.683 ^b^
Folic acid +	29 (93.5)	30 (93.8)	1.00 ^c^
Folic acid dose (mg)	5 (0–5)	5 (0–5)	0.982 ^b^
Calcium +	16 (51.6)	10 (31.3)	0.128 ^c^
Calcium dose (mg)	500 (0–1000)	0 (0–500)	0.065 ^b^
Methotrexate +	30 (96.8)	31 (96.9)	1.00 ^c^
Methotrexate dose (mg)	15 (0–25)	15 (0–25)	0.736 ^b^
Hydroxychloroquine +	11 (35.5)	4 (12.5)	0.04 * ^c^
Hydroxychloroquine dose (mg)	0 (0–300)	0 (0–300)	0.056 ^b^
Sulfasalazine +	21 (67.7)	24 (75)	0.585 ^c^
Sulfasalazine dose (mg)	1000 (0–3000)	1000 (0–3000)	0.747 ^b^

Abbreviations: BMI = Body Mass Index; CRP = C-reactive Protein; Anti-CCP = Anti-Cyclic Citrullinated Peptide Antibodies; RF = Rheumatoid Factor; HAQ = Health Assessment Questionnaire; ESR = Erythrocyte Sedimentation Rate; DAS28 = Disease Activity Score in 28 joints; VAS (Patient) = Visual Analog Scale for pain; PBMCs = peripheral blood mononuclear cells. ** Statistically significant value < 0.01; * Statistically significant value < 0.05; + Values represented as frequencies. ^a^ Student’s *t*-test; mean ± standard deviation. ^b^ Mann–Whitney U test; median (range). ^c^ Chi-square test; frequencies and percentages.

**Table 2:** Comparison of inflammatory biomarkers and *STAT4* gene expression between clinical activity groups.

Variable	Remission-Low Group (n = 31)	Moderate-High Group (n = 32)	*p*-Value
Inflammatory markers			
IL-12	87.2 (13.5–516.2)	103.2 (23.8–1920.4)	0.386 ^b^
IFN-ɣ	12.8 (0–1153)	12.9 (0–621.2)	0.815 ^b^
IL-23	12.3 (0–1247.5)	23 (0–1108.7)	0.353 ^b^
pSTAT4	0.15 ± 0.1	0.15 ± 0.1	0.814 ^a^
Underexpression +	19 (61.3)	14 (43.8)	0.210 ^c^
Overexpression +	12 (38.7)	18 (56.3)	
*STAT4* mRNA	0.63 (0.07–17.91)	0.63 (0.16–20.76)	0.929 ^b^
Underexpression +	19 (61.3)	20 (62.5)	0.921 ^c^
Overexpression +	12 (38.7)	12 (37.5)	

+ Values represented as frequencies. pSTAT4 = phosphorylated STAT4; IL-12 = interleukin-12; IFN-γ = interferon gamma; IL-23 = interleukin-23. ^a^ Student’s *t*-test; mean ± standard deviation. ^b^ Mann–Whitney U test; median (range). ^c^ Chi-square test; frequencies and percentages.

**Table 3:** Genotypic and allelic distribution of the rs7574865 and rs11889341 variants of the *STAT4* gene in patients with different levels of disease activity.

	Western Mexico				
	Remission-Low Group (n = 31)	Moderate-High Group (n = 32)	OR	95% CI	*p*-Value
*STAT4*: rs7574865 G/T					
GG ^1^	36.7 (11)	28.1 (9)	Reference		
GT ^2^	48.4 (15)	50 (16)	1.397	(0.449–4.35)	0.564
TT ^3^	16.7 (5)	21.9 (7)	2.512	(0.319–19.755)	0.381
G ^4^	60 (36)	53 (34)	Reference		
T ^5^	40 (24)	47 (30)	1.380	(0.961–1.981)	0.081
*STAT4*: rs11889341 C/T					
CC ^1^	36.7 (11)	28.1 (9)	Reference		
CT ^2^	48.4 (15)	50 (16)	1.397	(0.449–4.35)	0.564
TT ^3^	16.7 (5)	21.9 (7)	2.512	(0.319–19.755)	0.381
C ^4^	60 (36)	53 (34)	reference		
T ^5^	40 (24)	47 (30)	1.380	(0.961–1.981)	0.081

OR = odds ratio; CI = confidence interval. ^1^ Wild-type homozygote: rs7574865 G>T (GG) and rs11889341 C>T (CC). ^2^ Heterozygote genotype rs7574865 G>T (GT) and rs11889341 C>T (CT). ^3^ Variant homozygote rs7574865 G>T (TT) and rs11889341 C>T (TT). ^4^ Wild-type allele rs7574865 (G) and rs11889341 (C). ^5^ Variant allele rs7574865 (T) and rs11889341 (T).

**Table 4:** Linkage disequilibrium analysis and haplotype distribution for the rs7574865 and rs11889341 variants.

Western Mexico						
Haplotype	Remission-Low Group	Moderate-High Group	OR	95% CI	D′	r^2^
GC	60	53.12	1.00	-	1.000	1.000
TT	40	43.55	0.76	0.37–1.55		

Notes: OR = odds ratio; CI = confidence interval; D′ = linkage disequilibrium coefficient (association between alleles); r^2^ = correlation between loci.

## Data Availability

The data supporting the findings of this study are included in the article and its Supplementary Material. Further inquiries can be directed to the corresponding authors.

## Supplementary Materials

The following supporting information can be downloaded at: https://www.mdpi.com/article/10.3390/ijms262010011/s1.

## Abbreviations

The following abbreviations are used in this manuscript:

anti-CCP	Anti-cyclic citrullinated peptide antibodies
BMI	Body mass index
CI	Confidence interval
CRP	C-reactive protein
D′	Linkage disequilibrium
DAS28	Disease Activity Score in 28 joints
ESR	Erythrocyte sedimentation rate
*GWAS*	Genome-wide association studies
HAQ	Health Assessment Questionnaire
HCQ	Hydroxychloroquine
HLA	Human leukocyte antigen
IFN-γ	Interferon gamma
IL-12	Interleukin-12
IL-23	Interleukin-23
MCH	Mean corpuscular hemoglobin
MCV	Mean corpuscular volume
mRNA	Messenger RNA
MTX	Methotrexate
OR	Odds ratio
PBMCs	Peripheral blood mononuclear cells
PLT	Platelets
pSTAT4	Phosphorylated STAT4
r^2^	Correlation between loci
RA	Rheumatoid arthritis
RF	Rheumatoid factor
SSZ	Sulfasalazine
STAT4	Signal transducer and activator of transcription 4
VAS	Visual analogue scale
