# Supplementary material for: Genetic and Functional Characterization of STAT4 in Rheumatoid Arthritis Patients with Distinct Disease Activity
Source: Int J Mol Sci. 2025 Oct 15;26(20):10011. doi: 10.3390/ijms262010011 (PMC12562885; doi:10.3390/ijms262010011)
Supplement: Supplementary file 1 [file ijms-26-10011-s001.zip › ijms-3854188-supplementary.pdf]

Table S1. Clinical characteristics, medical history, anthropometric measurements, and hematological parameters in patients with rheumatoid arthritis.

Variable	Remission-Low Group (n=31)	Moderate-High Group (n=32)	p-value
Hematological parameters			
Erythrocytes (10^6/L)	4.71 ± 0.68	4.34 ± 0.35	<0.01** [a]
Neutrophils (%)	67.34 ± 7.9	65.63 ± 7.41	0.378 [a]
Lymphocytes (%)	24.90 (0-33.4)	27.3 (5.6-36.4)	0.496 [b]
Monocytes (%)	5.38 ± 1.85	5.93 ± 2.2	0.285 [a]
Eosinophils (%)	1.73 ± 1.28	1.34 ± 1.81	0.324 [a]
Hemoglobin (g/dL)	14.09 ± 2.04	12.63 ± 1.75	<0.01** [a]
Hematocrit (%)	42.13 ± 5.75	38.08 ± 4.36	<0.01** [a]
MCV (fL)	90.0 (67.6-102.3)	89.2 (70.7-98.9)	0.180 [b]
MCH (pg)	30.03 ± 3.04	29.04 ± 2.99	0.200 [a]
MCHC (g/dL)	33.44 ± 1.22	33.07 ± 1.32	0.262 [a]
Platelets (10^3/L)	339.52 ± 281.17	311.91 ± 162.87	0.637 [a]
MPV (fL)	9.8 (3.7-11.5)	9.7 (7.7-101)	0.983 [b]

MCV = Mean Corpuscular Volume; MCH = Mean Corpuscular Hemoglobin; MCHC = Mean Corpuscular Hemoglobin Concentration; MPV= Mean Platelet Volume. ** Statistically significant value < 0.01. a. Student's t-test; mean and standard deviation; b. Mann-Whitney U test; median and range.
